# Supplementary material for: Aedes-AI: Neural network models of mosquito abundance
Source: PLoS Comput Biol. 2021 Nov 19;17(11):e1009467. doi: 10.1371/journal.pcbi.1009467 (PMC8641871; doi:10.1371/journal.pcbi.1009467)
Supplement: S1 Appendix — (PDF) [file pcbi.1009467.s001.pdf]

S1 Appendix

Weather and MoLS Time Series

Fig A illustrates daily fluctuations and seasonal patterns observed in typical input weather data. MACA estimates of average daily temperature, precipitation, and relative humidity are plotted for two different climate models, GFDL-ESM2M [1] and CanESM2 [2].

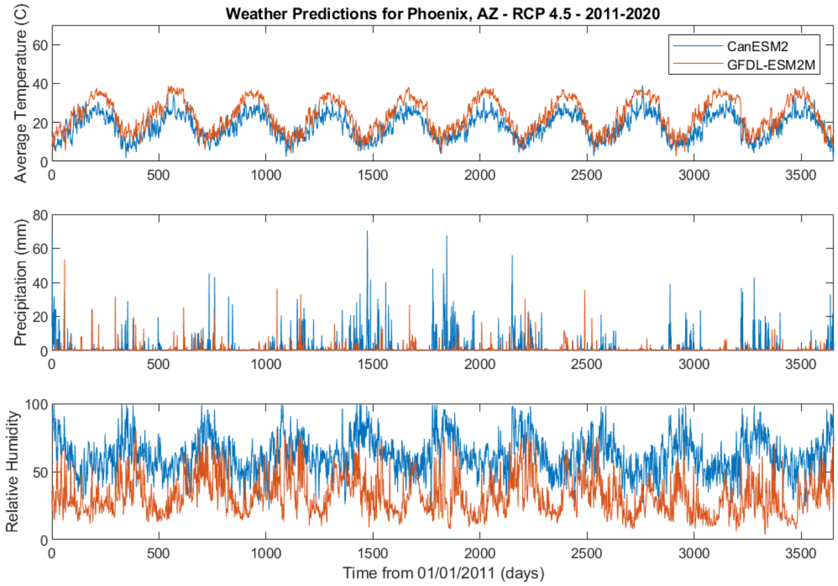

Fig A. MACA weather time series from 01/01/2011 to 12/31/2020 for Phoenix, AZ, based on two different climate models: GFDL-ESM2M and CanESM2. Top: average daily temperature; Middle: daily precipitation in millimeters; Bottom: relative humidity.

Visible differences between the two models lead to differences in MoLS predictions, as illustrated in Fig B: hotter and less humid conditions in the summer lead to double peaks in estimated mosquito abundance and to longer mosquito seasons.

MoLS output is sensitive to changes in location due to changes in associated weather data. Fig C shows MoLS predictions for Sacramento, CA (latitude: 38.56, longitude: -121.47) and for the centroid of Sacramento County (latitude: 38.35, longitude: -121.34). Note the differences in peak height (years 4, 5, 7, and 9) and in season length (years 1, 3, 4, and 10).

Fig D shows the correlation between the 2016 training and testing locations (*left* of the blue line), and 2016 training locations and capital cities (*right* of the blue line) for average temperature, precipitation, and MoLS predictions. The average temperature is seasonal, and thus, highly correlated among all locations, regardless of the relative temperature scale between locations. The low correlation for precipitation indicates that the ANN models are tested on samples with different weather features than the training samples. These differences in weather trends lead to moderately correlated annual MoLS predictions.

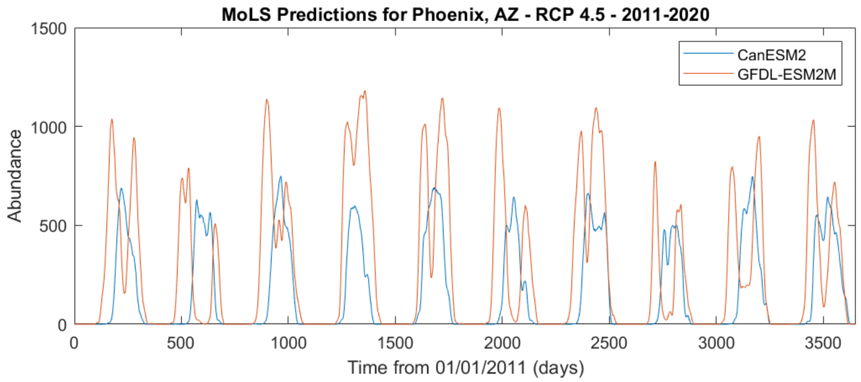

Fig B. Time series of gravid female abundance generated by MoLS using the weather data shown in Fig A.

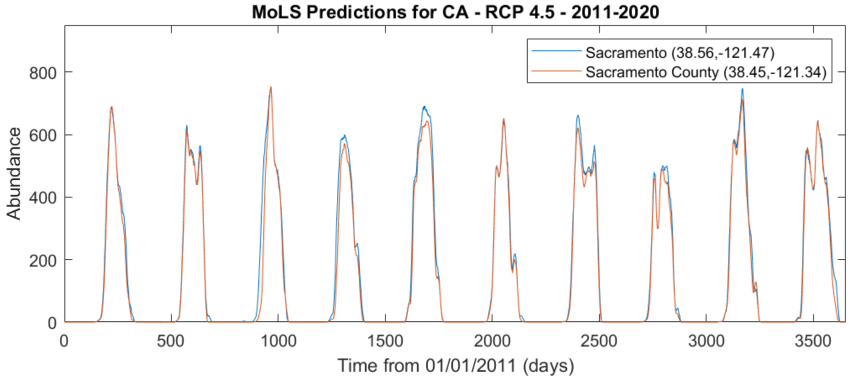

Fig C. Time series of gravid female abundance generated by MoLS for two different locations near Sacramento, CA.

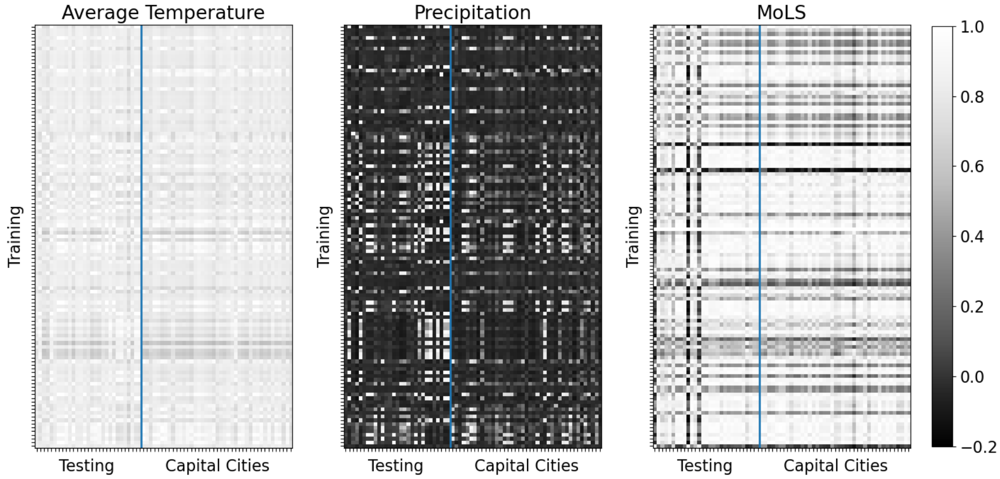

Fig D. Pearson correlation between training and testing data for average temperature, precipitation, and MoLS estimates in calendar year 2016. The correlations between training and testing locations are shown to the left of the blue line, and the correlations between training locations and capital cities are shown to the right. Each tick mark represents one location in the subset.

References

1. Geophysical Fluid Dynamics Laboratory ESM2M Model;. https://www.gfdl.noaa.gov/earth-system-model/.
2. The second generation Canadian Earth System Model (CanESM2);. https://www.canada.ca/en/environment-climate-change/services/climate-change/science-research-data/modeling-projections-analysis/centre-modelling-analysis/models/second-generation-earth-system-model.html.
