# Supplementary material for: Aedes-AI: Neural network models of mosquito abundance
Source: PLoS Comput Biol. 2021 Nov 19;17(11):e1009467. doi: 10.1371/journal.pcbi.1009467 (PMC8641871; doi:10.1371/journal.pcbi.1009467)
Supplement: S2 Appendix — (PDF) [file pcbi.1009467.s002.pdf]

S2 Appendix

Description of ANN Layers

This appendix provides additional details for the layers used in the neural network models described in Neural Network Models. Note that the parameters appearing in the weight matrices and kernels are learned during the training phase, as described in Model Training. Once trained, the models and their saved parameters may be used for any viable input sample. For a more thorough description of neural networks, we direct readers to [1]. Table A shows the variables used in the convolutional layers (ConvLayers), the LSTM layers, and the GRU layers. Each layer is described in more details below.

Variable	Description	Dimension/Value	Layers using Variable
l	Layer	—	All
$\boldsymbol{x}_l$	Input for layer l	$[n_l \times m_l]$	All
n_f	Number of filters	64	ConvLayers
f	Filter	$f \in [1, n_f]$	ConvLayers
$\boldsymbol{K}_f$	Kernel for filter f	$[3 \times m_l]$	ConvLayers
t	Time index of x_l	$t \in [0, n_l)$	All
$\boldsymbol{O}_l^C$	Output matrix for ConvLayer l	$[(n_l - 2) \times 64]$	ConvLayers
n_u	Length of hidden state	64	LSTM/GRU Layers
$\boldsymbol{c}_t$	Cell state at time t	$[1 \times n_u]$	LSTM Layers
$\boldsymbol{h}_t$	Hidden state at time t	$[1 \times n_u]$	LSTM/GRU Layers
$\boldsymbol{f}_t$	Forget gate at time t	$[1 \times n_u]$	LSTM Layers
$\boldsymbol{i}_t$	Input gate at time t	$[1 \times n_u]$	LSTM Layers
$\boldsymbol{o}_t$	Output gate at time t	$[1 \times n_u]$	LSTM Layers
$\boldsymbol{W}_*$	Weight matrix for gate $*$	$[(m_l + n_u) \times n_u]$	LSTM/GRU Layers
$\boldsymbol{b}_*$	Bias for gate $*$	$[1 \times n_u]$	LSTM/GRU Layers
$\boldsymbol{r}_t$	Reset gate at time t	$[1 \times n_u]$	GRU Layers
$\boldsymbol{z}_t$	Update gate at time t	$[1 \times n_u]$	GRU Layers

Table A. Variables used in the convolution layers, LSTM layers, and GRU layers. "All" means the variables were used in the three layer types.

Convolution Layers

Let $\boldsymbol{x}_l \in \mathbb{R}^{n_l \times m_l}$ be the input for layer l and $\boldsymbol{K}_f \in \mathbb{R}^{n_K \times m_l}$ be the kernel for filter $f \in [1, n_f]$. The dimensions of $\boldsymbol{x}_l$ are dependent on layer l, but the dimensions of $\boldsymbol{K}_f$ are constant for all filters f. In this article, $n_K = 3$. Let t be the time index of the input data such that $t \in [0, n_l)$. The output matrix $\boldsymbol{O}_l^C \in \mathbb{R}^{(n_l - n_K + 1) \times n_f}$ of convolution layer l is constructed as follows. For $t = 0, ..., (n_l - n_K)$ and $f = 1, ..., n_f$, let $\hat{\boldsymbol{x}} = \boldsymbol{x}_l[t : t + (n_K - 1), :] \in \mathbb{R}^{n_K \times m_l}$ (note that the t and $t + (n_K - 1)$ indices are inclusive). Then, $\boldsymbol{O}_l^C[t, f] = \sum_{a,b} [\boldsymbol{K}_f * \hat{\boldsymbol{x}}]_{a,b}$, where $*$ is component-wise multiplication, a ranges from 0 to $n_K - 1$, and b from 0 to $m_l - 1$. Table B shows the input and output dimensions for the convolution layers in the models discussed in Neural Network Models.

Layer	Input → Output Dimensions
ConvLayer 1	$[90 \times 4] \rightarrow [88 \times 64]$
ConvLayer 2	$[88 \times 64] \rightarrow [86 \times 64]$

Table B. Input/output dimensions of the convolution layers used in the models described in Neural Network Models.

LSTM Layers

The LSTM layers contain an LSTM unit with hidden state length $n_u = 64$ and have two sources of information flow: $\boldsymbol{C}_t \in \mathbb{R}^{1\times n_u}$, the cell state, which intuitively may be thought of as the long-term memory, and $\boldsymbol{h}_t \in \mathbb{R}^{1\times n_u}$, the hidden state, which is a filtered version of the cell state. Here $t \in [0, n_l)$, and $\boldsymbol{x}_l \in \mathbb{R}^{n_l \times m_l}$ is the input for the LSTM layer l. Two of the gates in the unit, the forget gate ($\boldsymbol{f}_t \in \mathbb{R}^{1\times n_u}$) and the input gate ($\boldsymbol{i}_t \in \mathbb{R}^{1\times n_u}$), selectively update the cell state. The third gate, the output gate ($\boldsymbol{o}_t \in \mathbb{R}^{1\times n_u}$), filters the cell state to produce the output, hidden state $\boldsymbol{h}_t$.

The above matrices are constructed as follows. First, the information from the previous hidden state, $\boldsymbol{h}_{t-1}$, is concatenated with the input at time t, $\boldsymbol{x}_t \in \mathbb{R}^{1\times m_l}$, to obtain $(\boldsymbol{x}_t \| \boldsymbol{h}_{t-1}) \in \mathbb{R}^{1\times(m_l+n_u)}$, where $(\cdot\|\cdot)$ is the concatenation operator. Next, the gates select the information from the concatenation $(\boldsymbol{x}_t \| \boldsymbol{h}_{t-1})$ used to update the cell and hidden states. Specifically, $\boldsymbol{f}_t = \sigma((\boldsymbol{x}_t \| \boldsymbol{h}_{t-1})\boldsymbol{W}_f + \boldsymbol{b}_f)$, $\boldsymbol{i}_t = \sigma((\boldsymbol{x}_t \| \boldsymbol{h}_{t-1})\boldsymbol{W}_i + \boldsymbol{b}_i)$, and $\boldsymbol{o}_t = \sigma((\boldsymbol{x}_t \| \boldsymbol{h}_{t-1})\boldsymbol{W}_o + \boldsymbol{b}_o)$, where σ is the sigmoid activation function and is applied entry-wise. Additionally, $\tilde{\boldsymbol{C}}_t$ represents potential information to add to the cell state: $\tilde{\boldsymbol{C}}_t = \tanh((\boldsymbol{x}_t \| \boldsymbol{h}_{t-1})\boldsymbol{W}_g + \boldsymbol{b}_g)$. Here, $\{\boldsymbol{W}_f, \boldsymbol{W}_i, \boldsymbol{W}_o, \boldsymbol{W}_g\} \in \mathbb{R}^{(m_l+n_u)\times n_u}$ are the weights of the three gates and potential cell state update, respectively, and $\{\boldsymbol{b}_f, \boldsymbol{b}_i, \boldsymbol{b}_o, \boldsymbol{b}_g\} \in \mathbb{R}^{1\times n_u}$ are the corresponding biases.

Finally, the cell state is updated based on the information from the forget gate and input gate: $\boldsymbol{C}_t = \boldsymbol{f}_t * \boldsymbol{C}_{t-1} + \boldsymbol{i}_t * \tilde{\boldsymbol{C}}_t$, and the hidden state is produced by filtering the cell state using the output gate: $\boldsymbol{h}_t = \tanh(\boldsymbol{C}_t) * \boldsymbol{o}_t$. Here again, $*$ denotes component-wise multiplication.

See Fig A *left* for a pictorial representation of the LSTM unit and Table C for the input and output dimensions for the LSTM layers in Neural Network Models. The input layer is the output of a Batch Normalization layer applied to the output of ConvLayer 2 from Table B. In LSTM Layer 1, the sequential hidden states, $\boldsymbol{h}_t$, are returned for each time step $t \in [0, n_l)$, whereas in LSTM Layer 2 only the final hidden state $\boldsymbol{h}_t$ is returned, where $t = n_l - 1$.

Layer	Input → Output Dimensions
LSTM Layer 1	$[86 \times 64] \rightarrow [86 \times 64]$
LSTM Layer 2	$[86 \times 64] \rightarrow [1 \times 64]$

Table C. Input/output dimensions of the LSTM layers used in Neural Network Models.

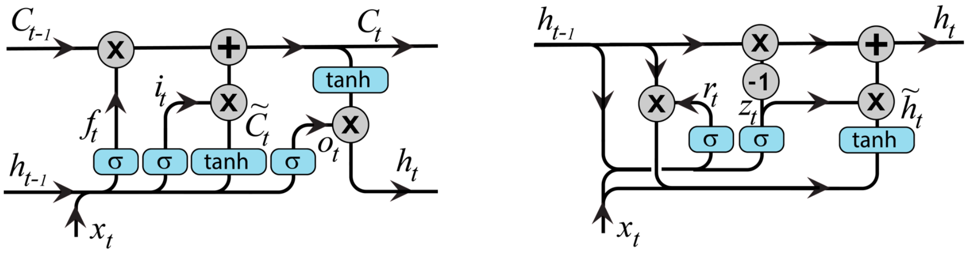

Fig A. The recurrent units in the LSTM (*left*) and GRU (*right*) layers.

GRU Layers

GRU layers each have a GRU unit with hidden state length $n_u = 64$ and gated structures to selectively allow information to propagate. In the GRU unit, $\boldsymbol{h}_t \in \mathbb{R}^{1\times n_u}$ represents the hidden state. The reset gate, $\boldsymbol{r}_t \in \mathbb{R}^{1\times n_u}$, controls the amount of previous information to include in the hidden state and the update gate, $\boldsymbol{z}_t \in \mathbb{R}^{1\times n_u}$, selectively adds new information to the hidden state.

Mathematically, the gates are structured similarly to the LSTM gates. First, the input at time step t, $\boldsymbol{x}_t \in \mathbb{R}^{1\times m_l}$ is concatenated with the previous hidden state, $(\boldsymbol{x}_t \| \boldsymbol{h}_{t-1}) \in \mathbb{R}^{1\times(m_l+n_u)}$. Next the gates select the information from the concatenation to be used to update the hidden state: $\boldsymbol{r}_t = \sigma((\boldsymbol{x}_t\|\boldsymbol{h}_{t-1})\boldsymbol{W}_r + \boldsymbol{b}_r)$ and $\boldsymbol{z}_t = \sigma((\boldsymbol{x}_t\|\boldsymbol{h}_{t-1})\boldsymbol{W}_z + \boldsymbol{b}_z)$ where $\{\boldsymbol{W}_r, \boldsymbol{W}_z\} \in \mathbb{R}^{(m_l+n_u)\times n_u}$ and $\{\boldsymbol{b}_r, \boldsymbol{b}_z\} \in \mathbb{R}^{1\times n_u}$ are the weights and biases of $\boldsymbol{r}_t$ and $\boldsymbol{z}_t$, respectively.

Next, $\tilde{\boldsymbol{h}}_t$ is created to represent the potential information to add to the hidden state: $\tilde{\boldsymbol{h}}_t = \tanh((\boldsymbol{x}_t\|(\boldsymbol{h}_{t-1} * \boldsymbol{r}_t))\boldsymbol{W}_h + \boldsymbol{b}_h)$ where $\boldsymbol{W}_h \in \mathbb{R}^{(m_l+n_u)\times n_u}$ and $\boldsymbol{b}_h \in \mathbb{R}^{1\times n_u}$ are the weight and biases of the potential hidden state, $\tilde{\boldsymbol{h}}_t$, and $*$ is component-wise multiplication. Finally, the hidden state is updated by weighting the potential information to add to the hidden state and the previous hidden state: $\boldsymbol{h}_t = \boldsymbol{z}_t * \tilde{\boldsymbol{h}}_t + (1 - \boldsymbol{z}_t) * \boldsymbol{h}_{t-1}$.

See Fig A *right* for a pictorial representation of the GRU unit and Table D for the input and output dimensions for the GRU layers in Neural Network Models. Like the LSTM layers, the input layer is the output of a Batch Normalization layer applied to the output of ConvLayer 2 from Table B. In GRU Layer 1 the sequential hidden states $\boldsymbol{h}_t$ are returned for $t \in [0, n_{x_l})$, whereas in GRU Layer 2 only the final hidden state $\boldsymbol{h}_t$ is returned for $t = n_{x_l} - 1$.

**Layer**	**Input → Output Dimensions**
GRU Layer 1	$[86 \times 64] \to [86 \times 64]$
GRU Layer 2	$[86 \times 64] \to [1 \times 64]$

Table D. Input/output dimensions of the GRU layers used in Neural Network Models.

Fully Connected Layers and Output Layers

For fully connected (FC) layer l, let $\boldsymbol{x}_l \in \mathbb{R}^{1\times(n_l\cdot m_l)}$ represent the input. Then the output of l, $\boldsymbol{O}_l^{FC} \in \mathbb{R}^{1\times n_{fc}}$, is: $\boldsymbol{O}_l^{FC} = \sigma(\boldsymbol{x}_l\boldsymbol{W} + \boldsymbol{b})$ for weight matrix $\boldsymbol{W} \in \mathbb{R}^{(n_l\cdot m_l)\times n_{fc}}$, bias $\boldsymbol{b} \in \mathbb{R}^{1\times n_{fc}}$, and specified output dimension $n_{fc} \in \mathbb{N}^+$, where σ is the ReLU activation function applied entry-wise.

In Model 1 (FF) we have two fully connected layers with $n_{fc} = 64$. Table E shows the input and output dimensions for the feed forward layers in Neural Network Models.

**Layer**	**Input → Output Dimensions**
FF 1	$[1 \times 5504] \to [1 \times 64]$
FF 2	$[1 \times 64] \to [1 \times 64]$

Table E. Input/output dimensions of the FC layers used in Neural Network Models.

The last layer for all three models is a fully connected output layer with $n_{fc} = 1$, shown in Table F.

**Layer**	**Input → Output Dimensions**
FC Output	$[1 \times 64] \to [1 \times 1]$

Table F. Input/output dimensions of the FC output layers used in Neural Network Models.

References

1. Goodfellow I, Bengio Y, Courville A. Deep Learning. vol. 1. MIT Press Massachusetts, USA:; 2017.
