# Supplementary material for: Aedes-AI: Neural network models of mosquito abundance
Source: PLoS Comput Biol. 2021 Nov 19;17(11):e1009467. doi: 10.1371/journal.pcbi.1009467 (PMC8641871; doi:10.1371/journal.pcbi.1009467)
Supplement: S3 Appendix — (PDF) [file pcbi.1009467.s003.pdf]

S3 Appendix

Performance Metrics

Global Performance Metrics

The four global fit performance metrics are defined as follows.

$$R^2_+ = \max\left(0, 1 - \frac{\sum_{i=1}^{n}(y_i - \hat{y}_i)^2}{\sum_{i=1}^{n}(y_i - \bar{y})^2}\right),$$

$$NRMSE = \frac{\sqrt{\sum_{i=1}^{n}\frac{1}{n}(\hat{y}_i - y_i)^2}}{y_{max} - y_{min}},$$

$$Rel.\ AUC\ Diff. = \frac{\sum_{i=2}^{n}\frac{1}{2}(y_{i-1} + y_i) - \sum_{i=2}^{n}\frac{1}{2}(\hat{y}_{i-1} + \hat{y}_i)}{\sum_{i=2}^{n}\frac{1}{2}(y_{i-1} + y_i)},$$

$$r = \frac{\sum_{i=1}^{n}(y_i - \bar{y})(\hat{y}_i - \hat{y})}{\sqrt{\sum_{i=1}^{n}(y_i - \bar{y})^2}\sqrt{\sum_{i=1}^{n}(\hat{y}_i - \hat{y})^2}},$$

where n is the output sample size, y_i represents the ith prediction by MoLS, $\bar{y}$ is the mean prediction by MoLS over the output sample, $\hat{y}_i$ is the ith prediction by the neural network model, $\hat{y}$ is the associated output sample mean, and $y_{max} - y_{min}$ is the range of the n MoLS predictions. We refer the reader to S5 Appendix for two examples of how these quantities reflect differences between abundance curves.

Season Feature Identification: Peak Timing and Season Length

In this section, we assume we are given an abundance time series $\{P_k\}_{k=1,...,n}$ (which could be the output of MoLS or of one of the ANNs) and describe how to identify regions where P_k consistently remains above a given threshold T.

We define the beginning of the range where P_k remains above T by finding the first day i such that P_i as well as the following 7 days, P_{i+1} to P_{i+7}, remain above the given threshold. We calculate a matching day j to mark the return to abundance values below T, which is defined as the first day $j > i$ such that P_j and abundance values in the next 7 days are lower than the threshold T.

Mathematically, i and j pairs are defined as follows:

$$i = \min_q \{q : P_{q+\ell} > T \text{ for } \ell = 0, \ldots, 7\}$$
$$j = \min_q \{q : P_{q+\ell} < T \text{ for } \ell = 0, \ldots, 7 \ \ \& \ \ q > i\}.$$

It is possible that there are multiple sections throughout the year where i and j pairs can be generated; this is particularly true for double-peak cities in Arizona and Southern California, where the *Aedes aegypti* population grows initially before dying off in the summer heat, rising again once the temperature cools. In these cases, there is a split season, and the pairs are enumerated as $(i_k, j_k)_T$ for $k = 1, \ldots, N$, where N is the number of regions above the threshold. Regions with $k = 2, \ldots, N$ are constrained such that i_k must occur after j_{k-1} ($j_{k-1} < i_k < j_k$).

Season Feature Performance Metrics

The metrics that quantify peak timing and season lengths are based on the difference, relative to MoLS data, in onset and offset times at various points in the mosquito season for a particular year. The pairs $(i_k, j_k)_T$ defined above for a specific threshold T are calculated for both the predicted (by the ANNs) and observed (MoLS) data on a yearly basis. We use the notation i_m and j_m to denote onset and offset days for the observed data and $\hat{\imath}_n$ and $\hat{\jmath}_n$ to denote onset and offset days for the predicted data. Each onset day i_m in the observed data is matched with the nearest onset day $\hat{\imath}_n$ in the predicted data, denoted $\hat{\imath}_n^m$, according to the minimum absolute difference of the points. This is mathematically defined as:

$$\hat{\imath}_n^m = \arg\min_{\hat{\imath}_n} |\hat{\imath}_n - i_m|.$$

Additionally, predicted days $\hat{\imath}_n$ are only allowed to match with a single observed day i_m. If a predicted day $\hat{\imath}_n$ would match with more than one observed day (i.e., there exists m_1, m_2 such that $\hat{\imath}_n^{m_1} = \hat{\imath}_n^{m_2}$), $\hat{\imath}_n$ is instead only matched to the day i_m with the minimum absolute difference, and the remaining observed days are matched with the remaining predicted days. This process is similarly done for the offset days j_m and $\hat{\jmath}_n$.

The resulting matchings are used to calculate onset (D_{on}) and offset (D_{off}) differences as follows

$$D_{on} = \hat{\imath}_n^m - i_m, \qquad D_{off} = \hat{\jmath}_n^m - j_m.$$

If D_{on} is negative, the ANN abundance exceeds the threshold T earlier than MoLS ($\hat{\imath}_n^m < i_m$), and if D_{on} is positive, the ANN abundance reaches T later ($\hat{\imath}_n^m > i_m$). This is similarly true for D_{off}.

Combined Performance Score

For each model and testing location combination, we define a fit-based performance metric

$$d = \left\langle \sqrt{M_1^2 + M_2^2} \right\rangle, \quad M_1 = 1 - \left(\bar{R}_+^2 \cdot \bar{r}\right)^{1/2}, \quad M_2 = \sqrt{(\overline{Rel.\ AUC\ Diff.})^2 + \overline{NRMSE}^2}$$

and a peak and seasonal performance metric

$$h = \frac{1}{Z}\Big[\sum_{i=1}^{N_{th}} (1 + \bar{p}_n(i)) \left(|\bar{D}_{on}^i| \cdot \sigma(D_{on}^i) + |\bar{D}_{off}^i| \cdot \sigma(D_{off}^i)\right)^{1/2}\Big],$$

where $\bar{*}$ and $\sigma(*)$ are the mean and standard deviation, respectively, of metric $*$ calculated over the testing years, $\bar{p}_n(i)$ is the average fraction of times the model did not reach the prescribed threshold, $Z = \sum_{i=1}^{N_{th}}(1 + \bar{p}_n(i))$ is a normalizing factor, and $N_{th} = 4$ is the number of thresholds. The mean $\langle\cdot\rangle$ in d is calculated over locations in the testing subset. The non-negative coefficient of determination and the Pearson correlation were combined into M_1 as a result of the strong correlation between R_+^2 and r. The use of terms of the form $|\bar{D}| \cdot \sigma(D)$ in h aims to penalize models with large values of $|\bar{D}_{on}|$ and/or $|\bar{D}_{off}|$ or large standard deviations. This indicates large departures from MoLS predictions, since all values of D_{on} and D_{off} are scaled to the average season length predicted by MoLS at the selected location. We see some instances where model predictions do not reach the threshold; this is particularly relevant for hot and dry locations at higher thresholds. To calculate $|\bar{D}|$ and $\sigma(D)$, we replace all instances where the model fails to meet a given threshold with the average of

$|D|$ for all location and years at the threshold. The $1 + \bar{p}_n(i)$ weight then penalizes models that do not always reach all of the prescribed thresholds.

The above information is combined into a single score S, which can be used to compare different models based on their performance on the testing subset:

$$S = \sqrt{S_1^2 + S_2^2}, \qquad S_1 = \frac{d}{\max(d)}, \qquad S_2 = \frac{h}{\max(h)},$$

where in each case, the maximum is taken over all combinations of model and location.
