# Supplementary material for: Aedes-AI: Neural network models of mosquito abundance
Source: PLoS Comput Biol. 2021 Nov 19;17(11):e1009467. doi: 10.1371/journal.pcbi.1009467 (PMC8641871; doi:10.1371/journal.pcbi.1009467)
Supplement: S4 Appendix — (PDF) [file pcbi.1009467.s004.pdf]

S4 Appendix

Seasonal feature analysis of all models

Here we provide additional results mentioned in Performance of Base Models, which support the conclusion that the variants improve on the base models, especially at higher threshold values.

Model	Metric	Threshold (% of Max MoLS Prediction)			
		**20%**	**40%**	**60%**	**80%**
FF	D_{on}	-0.006 ± 0.069	-0.013 ± 0.067	-0.036 ± 0.082	-0.044 ± 0.134
	D_{off}	0.019 ± 0.061	0.022 ± 0.067	0.039 ± 0.103	0.034 ± 0.135
LSTM	D_{on}	0.007 ± 0.065	-0.005 ± 0.062	-0.027 ± 0.07	-0.037 ± 0.088
	D_{off}	-0.001 ± 0.05	0.005 ± 0.079	0.01 ± 0.086	0.019 ± 0.088
GRU	D_{on}	-0.006 ± 0.057	-0.013 ± 0.059	-0.031 ± 0.082	-0.035 ± 0.098
	D_{off}	**-0.001 ± 0.044**	0.009 ± 0.072	0.01 ± 0.086	0.011 ± 0.096
FF HI	D_{on}	0.006 ± 0.065	-0.011 ± 0.064	-0.027 ± 0.069	-0.049 ± 0.105
	D_{off}	-0.008 ± 0.046	0.003 ± 0.055	0.012 ± 0.086	0.004 ± 0.099
FF LO	D_{on}	0.024 ± 0.07	0.007 ± 0.067	**-0.007 ± 0.07**	-0.031 ± 0.096
	D_{off}	-0.005 ± 0.045	-0.01 ± 0.051	0.005 ± 0.075	-0.008 ± 0.08
FF HI LO	D_{on}	**-0.001 ± 0.067**	-0.015 ± 0.063	-0.032 ± 0.076	-0.051 ± 0.12
	D_{off}	-0.004 ± 0.048	**0.002 ± 0.065**	0.013 ± 0.075	0.019 ± 0.093
LSTM HI	D_{on}	-0.003 ± 0.067	-0.018 ± 0.078	-0.027 ± 0.083	-0.034 ± 0.105
	D_{off}	-0.01 ± 0.051	-0.006 ± 0.076	-0.001 ± 0.091	0.002 ± 0.092
LSTM LO	D_{on}	0.002 ± 0.063	-0.011 ± 0.061	-0.02 ± 0.071	-0.031 ± 0.109
	D_{off}	0.007 ± 0.06	0.016 ± 0.087	0.02 ± 0.091	0.023 ± 0.125
LSTM HI LO	D_{on}	0.017 ± 0.072	**-0.003 ± 0.083**	-0.018 ± 0.094	-0.024 ± 0.108
	D_{off}	-0.012 ± 0.047	-0.008 ± 0.071	-0.003 ± 0.1	0.004 ± 0.11
GRU HI	D_{on}	-0.003 ± 0.058	-0.014 ± 0.068	-0.022 ± 0.075	**-0.028 ± 0.088**
	D_{off}	-0.002 ± 0.052	-0.006 ± 0.062	**0.0 ± 0.059**	0.001 ± 0.092
GRU LO	D_{on}	-0.005 ± 0.06	-0.016 ± 0.063	-0.031 ± 0.074	-0.038 ± 0.098
	D_{off}	-0.003 ± 0.044	0.005 ± 0.057	0.007 ± 0.071	0.016 ± 0.095
GRU HI LO	D_{on}	-0.007 ± 0.067	-0.022 ± 0.067	-0.038 ± 0.091	-0.04 ± 0.092
	D_{off}	0.011 ± 0.05	0.009 ± 0.07	0.013 ± 0.104	**0.0 ± 0.102**

Table A. Seasonal feature metrics for the testing subset. The double line separates the base models (top three rows) from the variant models (below). Seasonal differences for a location are scaled by the average length of the season at the 20% threshold. Entries are formatted as $\bar{D} \pm \sigma(D)$ and bold entries correspond to the lowest values of $|\bar{D}| \cdot \sigma(D)$ for each threshold, with $D = D_{on}$ or D_{off}. See S3 Appendix for a description of the metrics.
