# Supplementary material for: Aedes-AI: Neural network models of mosquito abundance
Source: PLoS Comput Biol. 2021 Nov 19;17(11):e1009467. doi: 10.1371/journal.pcbi.1009467 (PMC8641871; doi:10.1371/journal.pcbi.1009467)
Supplement: S5 Appendix — (PDF) [file pcbi.1009467.s005.pdf]

S5 Appendix

Case Study

We present an analysis of the base GRU and GRU variant models for Avondale, Arizona and Collier County, Florida to illustrate the differences in performance observed among the model archetypes and across different climate regions. Figs A and B compare the MoLS abundance to the estimates of the ANN models for the year 2020. Abundance curves are on the left and associated global fit metrics are on the right. Such a juxtaposition brings visual context to differences in metric values and trends observed in the main sections of the article.

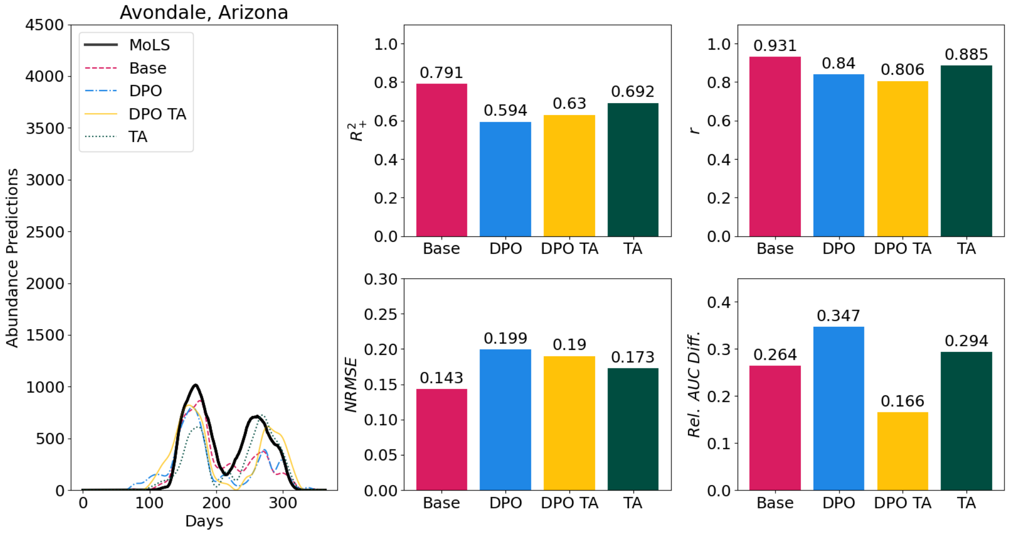

Fig A. The 2020 abundance curves for the GRU model variants and associated performance metrics for Avondale, Arizona. The reader is referred to S3 Appendix for descriptions of the metrics.

In Collier County we see one long season, whereas in Avondale there are two distinct peaks. When compared to the base model, the HI and LO variants lead to improved performance in both regions, with lower $Rel.\ AUC\ Diff.$ values for the HI model and better $NRMSE$ for the LO model. In particular, the HI variant is able to capture MoLS low abundance estimates during the hot summer months in Arizona (left panel of Fig A). On the other hand, the global fit performance of the HI LO model is comparable to or worse than that of the base model. Table A displays the associated seasonal metrics values, indicating slightly more consistent performance for the HI version, which achieves lowest values of $\max(|D_{on}|, |D_{off}|)$ (less than 0.048 in Avondale, Arizona and less than 0.036 in Collier County, Florida).

In summary, although all models are able to reproduce the trends observed in MoLS abundance curves in Florida, the HI version appears to better capture the dip in abundance due to hot summer months in Arizona, without deterioration of performance in other parts of the year.

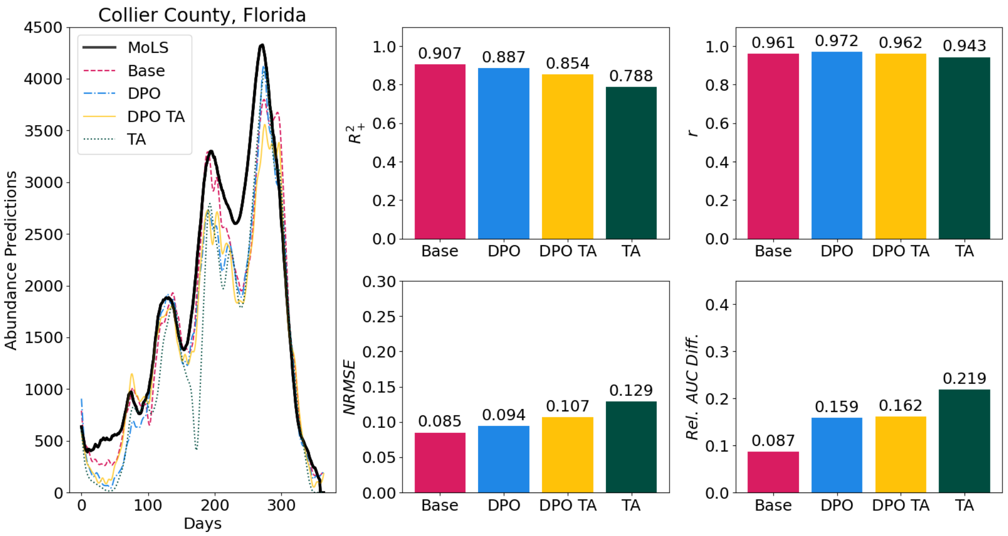

Fig B. The 2020 abundance curves for the GRU model variants and associated performance metrics for Collier County, Florida. The reader is referred to S3 Appendix for descriptions of the metrics.

**Model**	**Metric**	**Threshold (% of Max MoLS Prediction)**			
		**20%**	**40%**	**60%**	**80%**
Base	D_{on}	0.012	-0.018	0.0	-0.042
	D_{off}	0.024	0.073	-0.012	-0.024
HI	D_{on}	0.0	-0.036	-0.048	-0.06
	D_{off}	0.006	0.009	-0.009	-0.036
LO	D_{on}	0.0	-0.024	-0.018	0.0
	D_{off}	-0.006	-0.057	-0.012	-0.024
HI LO	D_{on}	-0.03	-0.021	-0.021	-0.024
	D_{off}	0.012	-0.066	-0.009	-0.03

(a) 2020 Avondale, Arizona. Season length $\ell_S = 165.5$ days

**Model**	**Metric**	**Threshold (% of Max MoLS Prediction)**			
		**20%**	**40%**	**60%**	**80%**
Base	D_{on}	-0.043	-0.023	-0.022	-0.047
	D_{off}	-0.018	-0.009	-0.004	0.011
HI	D_{on}	-0.02	-0.013	-0.014	-0.04
	D_{off}	-0.016	-0.02	-0.014	-0.036
LO	D_{on}	-0.051	-0.007	0.004	-0.029
	D_{off}	-0.018	-0.033	-0.004	-0.007
HI LO	D_{on}	-0.04	-0.011	-0.018	-0.036
	D_{off}	-0.011	-0.025	-0.014	-0.04

(b) 2020 Collier County, Florida. Season length $\ell_S = 276.6$ days

Table A. Season feature metrics for (a) Avondale, Arizona and (b) Collier County, Florida. Seasonal differences for a location and year are scaled by the average length of the season at the 20% threshold. See S3 Appendix for a description of the metrics.
