# Supplementary material for: Aedes-AI: Neural network models of mosquito abundance
Source: PLoS Comput Biol. 2021 Nov 19;17(11):e1009467. doi: 10.1371/journal.pcbi.1009467 (PMC8641871; doi:10.1371/journal.pcbi.1009467)
Supplement: S6 Appendix — (PDF) [file pcbi.1009467.s006.pdf]

S6 Appendix

ANN Variations

Table A shows global fit metrics (means and standard deviations are calculated over all years and locations in the testing subset) for the three main models and the following variations. The prime version is trained in the same way as the base version but with a different set of randomly chosen training samples. The 2000 version of each model uses 2000 instead of 1000 randomly chosen samples per training location. Finally, the $\Delta 120$ version uses $\Delta = 120$ days of weather data instead of $\Delta = 90$ days. Recall that all of these models take Δ consecutive days of weather time series as input, to estimate abundance on the Δth day. Table B shows similar comparisons for the seasonal metrics.

**FF Variants**				
**Model**	**Metric**			
	R^2_+	$NRMSE$	*Rel. AUC Diff.*	r
FF	0.871 ± 0.142	0.112 ± 0.073	0.09 ± 0.239	0.961 ± 0.038
FF′	0.901 ± 0.127	0.096 ± 0.074	0.042 ± 0.234	0.969 ± 0.033
FF_{2000}	0.898 ± 0.141	0.095 ± 0.072	0.037 ± 0.223	0.967 ± 0.041
$\text{FF}_{\Delta 120}$	0.894 ± 0.14	0.097 ± 0.069	0.015 ± 0.212	0.966 ± 0.037
**LSTM Variants**				
**Model**	**Metric**			
	R^2_+	$NRMSE$	*Rel. AUC Diff.*	r
LSTM	0.916 ± 0.118	0.088 ± 0.07	0.031 ± 0.224	0.974 ± 0.028
LSTM′	0.91 ± 0.129	0.089 ± 0.065	0.06 ± 0.196	0.971 ± 0.035
LSTM_{2000}	0.919 ± 0.126	0.084 ± 0.071	0.017 ± 0.22	0.974 ± 0.029
$\text{LSTM}_{\Delta 120}$	0.904 ± 0.131	0.092 ± 0.075	-0.019 ± 0.219	0.97 ± 0.037
**GRU Variants**				
**Model**	**Metric**			
	R^2_+	$NRMSE$	*Rel. AUC Diff.*	r
GRU	0.923 ± 0.119	0.083 ± 0.071	0.036 ± 0.207	0.975 ± 0.029
GRU′	0.915 ± 0.146	0.084 ± 0.068	-0.055 ± 0.207	0.975 ± 0.026
GRU_{2000}	0.921 ± 0.118	0.083 ± 0.06	0.04 ± 0.185	0.975 ± 0.029
$\text{GRU}_{\Delta 120}$	0.921 ± 0.109	0.084 ± 0.052	0.002 ± 0.157	0.973 ± 0.029

Table A. Global performance metrics for variations of the base models on the testing subset.

The results are highly consistent for each metric, with the mean score of each variation falling within one standard deviation of the mean scores obtained by variations of the same model. This suggests that the default choices for Δ (90 days) and for the size of the training set (1000 samples per location for the base versions), lead to models whose performance is representative of what ANNs trained on MoLS can accomplish. In addition, the variability observed in the means displayed in these tables provides intuitive context for deciding when a model performs better than another, given that each model performance is a random variable that depends on the samples randomly selected during the training process.

**FF Variants**					
**Model**	**Metric**	**Threshold (% of Max MoLS Prediction)**			
		**20%**	**40%**	**60%**	**80%**
FF	D_{on}	-0.006 ± 0.069	-0.013 ± 0.067	-0.036 ± 0.082	-0.044 ± 0.134
	D_{off}	0.019 ± 0.061	0.022 ± 0.067	0.039 ± 0.103	0.034 ± 0.135
FF′	D_{on}	0.003 ± 0.074	-0.011 ± 0.068	-0.03 ± 0.079	-0.058 ± 0.11
	D_{off}	-0.006 ± 0.042	0.0 ± 0.044	0.018 ± 0.074	0.022 ± 0.089
FF_{2000}	D_{on}	-0.01 ± 0.064	-0.019 ± 0.064	-0.03 ± 0.066	-0.049 ± 0.1
	D_{off}	-0.005 ± 0.041	0.003 ± 0.057	0.013 ± 0.064	0.013 ± 0.098
$FF_{\Delta 120}$	D_{on}	-0.007 ± 0.07	-0.022 ± 0.081	-0.032 ± 0.083	-0.052 ± 0.107
	D_{off}	-0.017 ± 0.041	-0.013 ± 0.067	-0.006 ± 0.087	-0.008 ± 0.102
**LSTM Variants**					
**Model**	**Metric**	**Threshold (% of Max MoLS Prediction)**			
		**20%**	**40%**	**60%**	**80%**
LSTM	D_{on}	0.007 ± 0.065	-0.005 ± 0.062	-0.027 ± 0.07	-0.037 ± 0.088
	D_{off}	-0.001 ± 0.05	0.005 ± 0.079	0.01 ± 0.086	0.019 ± 0.088
LSTM′	D_{on}	-0.014 ± 0.06	-0.021 ± 0.056	-0.029 ± 0.076	-0.036 ± 0.1
	D_{off}	0.007 ± 0.052	0.012 ± 0.083	0.021 ± 0.095	0.024 ± 0.114
$LSTM_{2000}$	D_{on}	0.002 ± 0.062	-0.01 ± 0.063	-0.024 ± 0.086	-0.035 ± 0.092
	D_{off}	-0.009 ± 0.041	-0.004 ± 0.067	-0.001 ± 0.074	0.011 ± 0.089
$LSTM_{\Delta 120}$	D_{on}	0.007 ± 0.072	-0.005 ± 0.074	-0.015 ± 0.078	-0.014 ± 0.098
	D_{off}	-0.016 ± 0.05	-0.016 ± 0.06	-0.009 ± 0.072	-0.0 ± 0.103
**GRU Variants**					
**Model**	**Metric**	**Threshold (% of Max MoLS Prediction)**			
		**20%**	**40%**	**60%**	**80%**
GRU	D_{on}	-0.006 ± 0.057	-0.013 ± 0.059	-0.031 ± 0.082	-0.035 ± 0.098
	D_{off}	-0.001 ± 0.044	0.009 ± 0.072	0.01 ± 0.086	0.011 ± 0.096
GRU′	D_{on}	0.042 ± 0.074	0.024 ± 0.073	0.002 ± 0.075	-0.014 ± 0.086
	D_{off}	-0.015 ± 0.041	-0.02 ± 0.057	-0.016 ± 0.051	-0.01 ± 0.083
GRU_{2000}	D_{on}	-0.007 ± 0.061	-0.015 ± 0.061	-0.029 ± 0.073	-0.039 ± 0.108
	D_{off}	-0.006 ± 0.05	-0.003 ± 0.057	0.006 ± 0.06	0.006 ± 0.098
$GRU_{\Delta 120}$	D_{on}	-0.014 ± 0.059	-0.018 ± 0.062	-0.026 ± 0.07	-0.024 ± 0.088
	D_{off}	-0.009 ± 0.053	-0.017 ± 0.05	-0.022 ± 0.062	-0.029 ± 0.094

Table B. Seasonal fit metrics for variations of the base models on the testing subset.
