# Supplementary material for: Aedes-AI: Neural network models of mosquito abundance
Source: PLoS Comput Biol. 2021 Nov 19;17(11):e1009467. doi: 10.1371/journal.pcbi.1009467 (PMC8641871; doi:10.1371/journal.pcbi.1009467)
Supplement: S7 Appendix — (PDF) [file pcbi.1009467.s007.pdf]

S7 Appendix

Locations used in the principal data set for training, validation, and testing

State	Training/Validation Locations
Arizona (Cities)	Anthem*, Apache Junction*, Buckeye*, Bullhead City*, Casa Grande*, Casas Adobes, Catalina Foothills, Chandler*, Douglas, Drexel Heights, El Mirage*, Flagstaff, Florence*, Flowing Wells, Fountain Hills*, Gilbert*, Glendale*, Goodyear*, Green Valley, Kingman, Lake Havasu City*, Mesa*, New River*†, Oro Valley†, Paradise Valley*, Payson, Peoria*, Phoenix*, Prescott†, Queen Creek*, Rio Rico†, Sahuarita, San Luis*, San Tan Valley*, Scottsdale*, Sierra Vista, Somerton*, Sun City*, Surprise*, Tanque Verde, Tempe*, Yuma*
California (Counties)	Alameda, Contra Costa, Fresno, Glenn, Imperial*, Inyo, Kern, Kings, Lake, Los Angeles, Madera, Marin, Merced, Mono†, Monterey†, Napa†, Orange†, Placer, Sacramento†, San Benito, San Bernardino*, San Diego, San Joaquin, San Luis Obispo, San Mateo, Santa Barbara, Santa Clara, Santa Cruz, Solano, Sonoma, Sutter, Tulare
Connecticut (Counties)	New Haven†
Florida (Counties)	Calhoun, Escambia, Gadsden, Hillsborough, Holmes, Jefferson, Lee, Liberty, Madison, Manatee, Martin, Miami-Dade, Okaloosa†, Pasco, Polk, Santa Rosa, St. Johns, Taylor, Wakulla, Walton, Washington
New Jersey (Counties)	Cumberland, Mercer, Monmouth†, Morris†, Sussex, Warren
New York (Counties)	Bronx†, Kings†, Nassau†, Queens, Rockland†, Westchester
North Carolina (Counties)	New Hanover†, Transylvania, Wake
Texas (Counties)	Hidalgo, Tarrant
Wisconsin (Counties)	Dane†, Milwaukee†

Table A. Training and validation locations. ∗ locations are used for high temperature oversampling and † are used for low temperature oversampling. States and locations are organized alphabetically.

State	Testing Locations
Florida (Counties)	Osceola, Pinellas, Collier, Jackson
Texas (Counties)	Cameron
New Jersey (Counties)	Salem, Essex
New York (Counties)	New York, Richmond
North Carolina (Counties)	Forsyth, Pitt
Connecticut (Counties)	Fairfield
Arizona (Cities)	Fortuna Foothills, Prescott Valley, Maricopa, Nogalees, Eloy, Marana, Tucson, Avondale
California (Counties)	Riverside, Ventura, Shasta, Yuba, Butte, Stanislaus, Yolo, Colusa
Wisconsin (Counties)	Waukesha

Table B. Testing locations. States are organized bottom to top by their performance on the combined metric and locations within each state are organized left to right in the order they appear in Fig 8.
