# Supplementary material for: Aedes-AI: Neural network models of mosquito abundance
Source: PLoS Comput Biol. 2021 Nov 19;17(11):e1009467. doi: 10.1371/journal.pcbi.1009467 (PMC8641871; doi:10.1371/journal.pcbi.1009467)
Supplement: S8 Appendix — (PDF) [file pcbi.1009467.s008.pdf]

S8 Appendix

Capital Cities Data Set

This appendix illustrates the performance of the GRU HI model on the Capital Cities dataset. It includes 44 locations, corresponding to all capital cities in the contiguous US (48 states plus DC) situated in counties that were not used for model training, validation, or testing. Phoenix, Sacramento, Trenton, Raleigh, and Madison are therefore omitted. Results for these or nearby locations are available in the main text if they were used for testing. The weather data are the MACA time series [1–4] for the Canadian Can-ESM2 [5] model under the RCP4.5 scenario.

Fig A displays box plots for the four global metrics assessing how the ANN estimates compare to those of MoLS, over nine years (2012-2020) of weather data. We see that the GRU HI model replicates MoLS time series very well, both in trend and size, except for a few outliers listed in Table A.

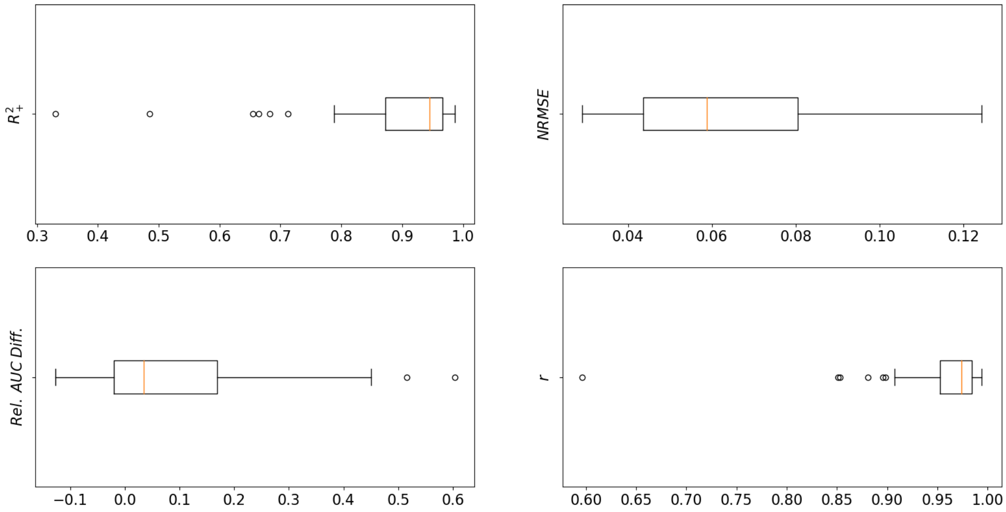

Fig A. Global performance metrics for the GRU HI model on the Capital Cities data set. The metrics are calculated over all years, so each data point represents the metric for one capital city from 2012-2020.

**Metric**	**Outliers**
R^2	Idaho, Nevada, North Dakota, Oregon, Vermont, Wyoming
$Rel.\ AUC\ Diff.$	Oregon, Wyoming
r	Idaho, Nevada, North Dakota, Oregon, Vermont, Wyoming

Table A. Capital city outliers in the global performance box plots, Fig A.

The D_{on} and D_{off} metrics for seasonal fit show good performance as well, Fig B, but with a larger number of outliers (see Table B), which now include Arkansas, Colorado, Indiana, Maine, Montana, New Hamshire, New Mexico, Utah, and Washington State. The number of outliers decreases as the threshold increases, suggesting the GRU HI model does not reach the 80% threshold in some of the poor performing locations. This is captured in the combined score, Fig C.

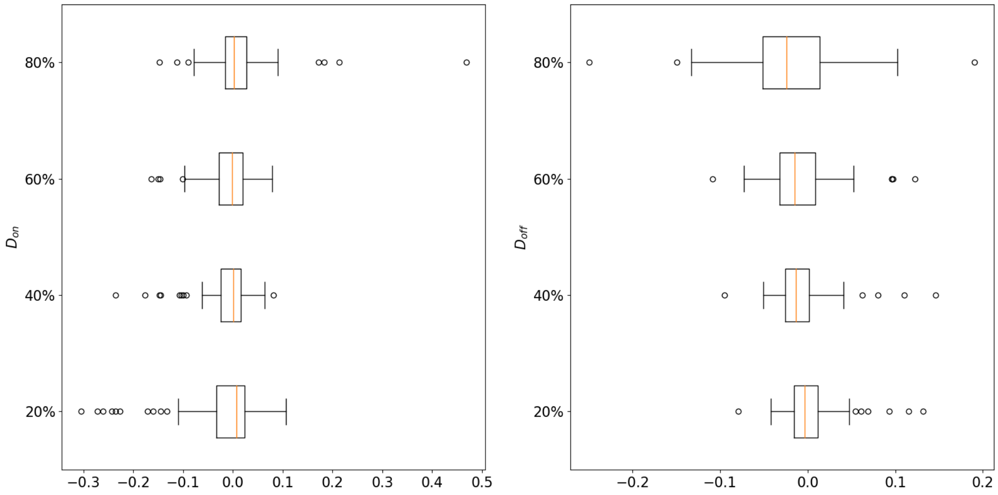

Fig B. Seasonal fit metrics for the GRU HI model on the Capital Cities data set. The metrics are averaged over all years, so each data point represents the average value for one capital city.

Metric	Threshold	Outliers
D_{on}	20%	Maine, Montana, Nevada, New Hampshire, New Mexico, North Dakota, Oregon, Vermont, Washington, Wyoming
	40%	Idaho, Indiana, Maine, Montana, New Hampshire, New Mexico, Oregon, Vermont, Wyoming
	60%	Colorado, Maine, New Mexico, Wyoming
	80%	Arkansas, Maine, Montana, Nevada, North Dakota, Oregon, Washington
D_{off}	20%	Idaho, Montana, Nevada, North Dakota, Oregon, Utah, Wyoming
	40%	Idaho, Nevada, Oregon, Vermont, Wyoming
	60%	Idaho, New Mexico, Oregon, Utah, Vermont
	80%	North Dakota, Oregon, Vermont

Table B. Capital city outliers in the seasonal performance box plots, Fig B.

We observe a strong correlation between the ANN performance and the number of mosquitoes predicted by MoLS: performance is lower in regions where expected mosquito numbers are lower overall. This is illustrated in Fig C. The main map shows the combined score (defined in S3 Appendix) of the GRU HI model. Each state is colored according to the performance of the ANN on weather data for its capital city, with yellow indicating high performance (low score) and purple low performance (score of 1 or higher). States colored in gray are those not included in the Capital Cities dataset. The inset shows a similar map, but now with colors indicating MoLS average abundance for the years in the dataset (2012-2020). Dark colors correspond to very low average abundance (less than 20 mosquitoes per day) while light colors indicate average abundance over 800 mosquitoes per day. Both scales are logarithmic. The log scale correlation (Pearson coefficient $r = -0.913$) is evident visually and supports the conclusion that the GRU HI model is a good MoLS replacement in regions of high mosquito abundance.

To illustrate how good and bad performance scores translate into errors on mosquito estimates, Fig D plots the abundance time series generated by MoLS (black solid curves) and by the GRU HI model (red dashed curves) in locations of low (Nevada and

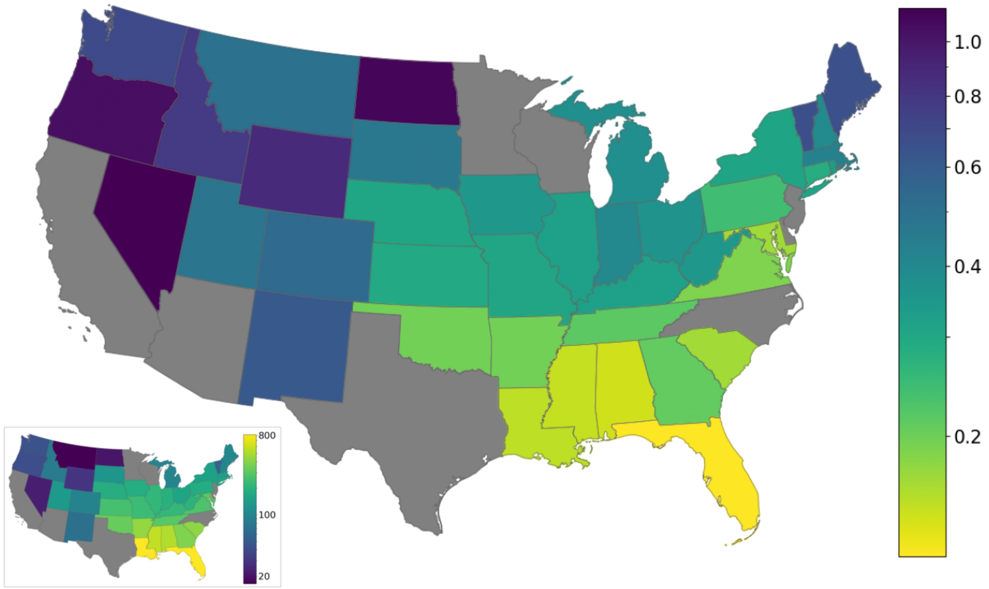

Fig C. Combined scores on capital cities (see S3 Appendix for metric definition). Each state is colored according to the corresponding capital city score. The inset shows the average daily number of mosquitoes predicted by MoLS for each capital city; the log scale ranges from 16.44 (dark purple) to 814.28 (yellow). States in gray were omitted since the capital city was part of either the training or the testing subsets. We see a strong correlation between score and number of mosquitoes.
Base map obtained from the United States Census Bureau (`https://www.census.gov/geographies/mapping-files/time-series/geo/carto-boundary-file.html`).

Montana), medium (Kentucky and Michigan), and high (Georgia and Louisiana) performance based on their combined scores.

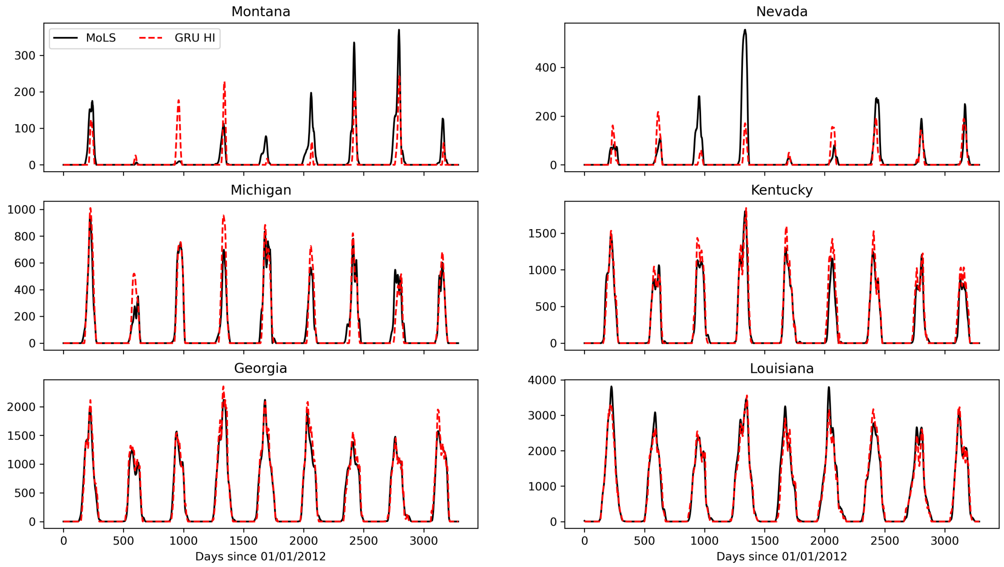

Fig D. Comparison of the GRU HI output to MoLS data for capital cities of varying scores (Fig C).

References

1. MACA datasets;. http://www.climatologylab.org/maca.html. The climate forcings in the MACAv2-METDATA were drawn from a statistical downscaling of global climate model (GCM) data from the Coupled Model Intercomparison Project 5 [2] utilizing the Multivariate Adaptive Constructed Analogs [3] method with the METDATA [4] observational dataset as training data.

2. Taylor KE, Stouffer RJ, Meehl GA. An overview of CMIP5 and the experiment design. Bulletin of the American Meteorological Society. 2012;93(4):485–498.

3. Abatzoglou JT, Brown TJ. A comparison of statistical downscaling methods suited for wildfire applications. International Journal of Climatology. 2012;32(5):772–780.

4. Abatzoglou JT. Development of gridded surface meteorological data for ecological applications and modelling. International Journal of Climatology. 2013;33(1):121–131.

5. The second generation Canadian Earth System Model (CanESM2);. https://www.canada.ca/en/environment-climate-change/services/climate-change/science-research-data/modeling-projections-analysis/centre-modelling-analysis/models/second-generation-earth-system-model.html.
