# Supplementary material for: Aedes-AI: Neural network models of mosquito abundance
Source: PLoS Comput Biol. 2021 Nov 19;17(11):e1009467. doi: 10.1371/journal.pcbi.1009467 (PMC8641871; doi:10.1371/journal.pcbi.1009467)
Supplement: S9 Appendix — (PDF) [file pcbi.1009467.s009.pdf]

S9 Appendix

MoLS-GRU HI Comparison

Figs A - E show how the output of the GRU HI model compares to MoLS data over a period of 9 years (2012-2020). Each panel corresponds to one column of Fig 8 and the locations are in the same order as in Fig 8.

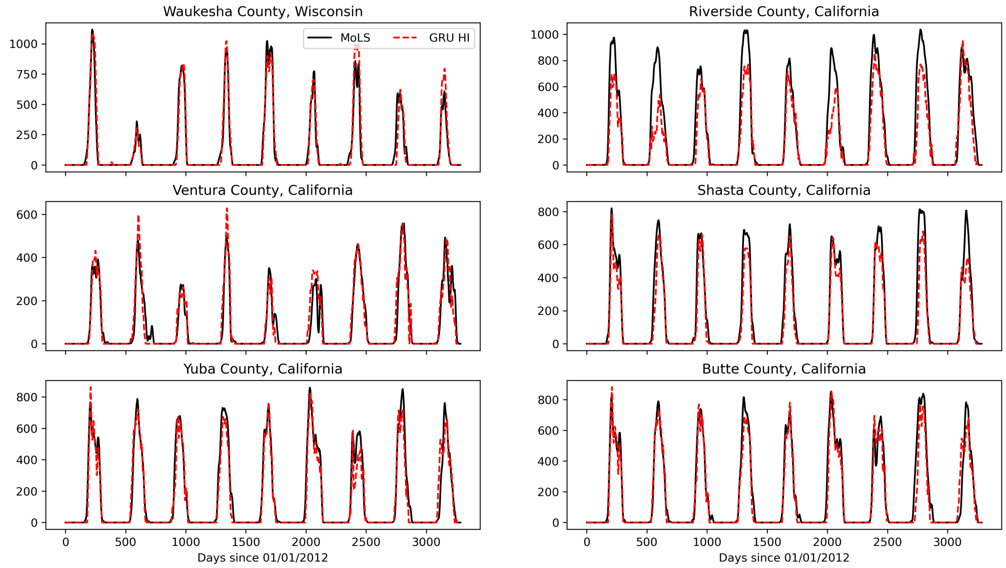

Fig A. Comparison of the GRU HI model to MoLS data for testing locations in Wisconsin and California.

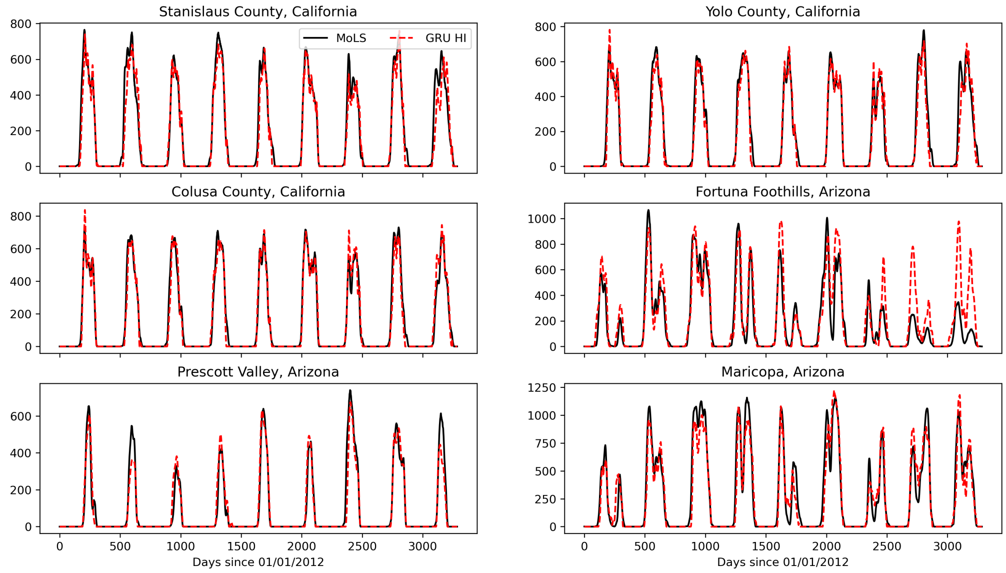

Fig B. Comparison of the GRU HI model to MoLS data for testing locations in California and Arizona.

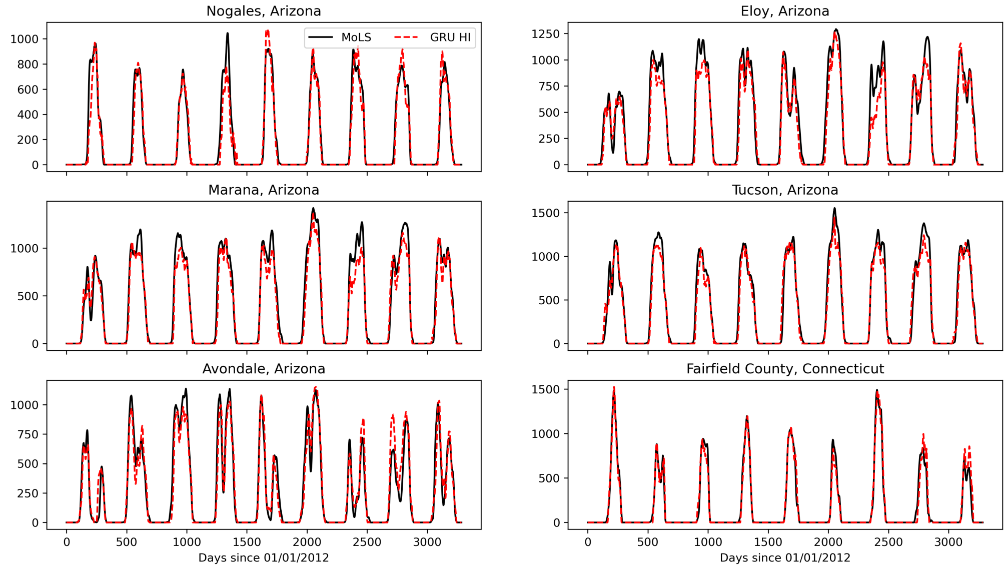

Fig C. Comparison of the GRU HI model to MoLS data for testing locations in Arizona and Connecticut.

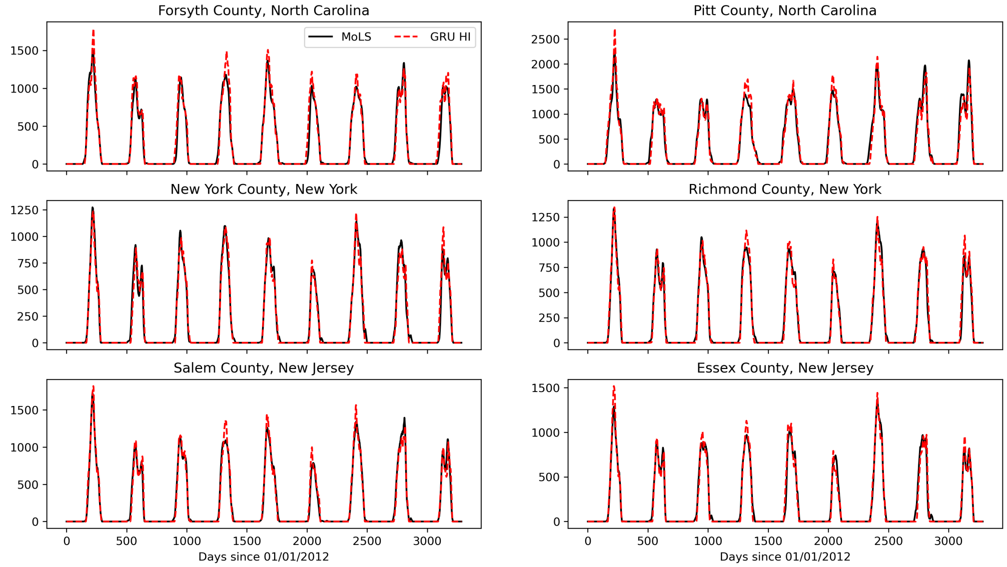

Fig D. Comparison of the GRU HI model to MoLS data for testing locations in North Carolina, New York, and New Jersey.

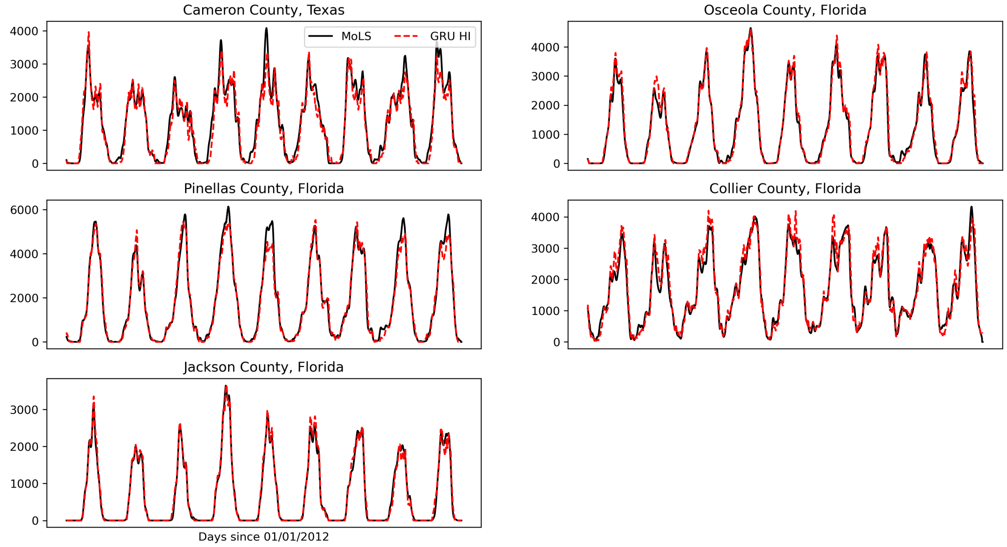

Fig E. Comparison of the GRU HI model to MoLS data for testing locations in Texas and Florida.
